# Frequency of dipeptides and antidipeptides

**DOI:** 10.5936/csbj.201308001

**Published:** 2013-08-14

**Authors:** Oliviero Carugo

**Affiliations:** a Department of Chemistry, University of Pavia, viale Taramelli12, I-27100 Pavia, Italy; b Department of Structural and Computational Biology, Max F. Perutz Laboratories, Vienna University, Campus Vienna Biocenter 5, A-1030 Vienna, Austria

**Keywords:** Protein sequence, Dipeptide, Amino acid composition, Antidipeptide

## Abstract

Although it is reasonable to expect that the frequency of a generic dipeptide XY in proteins is the same of its counterpart YX, on the basis of an accurate statistical analysis of a large number of protein sequences, it appears that some dipeptides XY are considerably more frequent than their mirror images YX, referred to as *antidipeptides*. Given that it has been verified that this unexpected anisotropic frequency of occurrence is unbiased by the type of protein sequences that are analyzed, it is possible to conclude that this is a genuine phenomenon. Nevertheless, it was impossible to find the mechanism underlying this unexpected phenomenon, which does not seem to be related to diverse conformational propensities, to the different conformational flexibility of the peptide/antidipeptide pair, to dissimilar accessibility to the solvent or to gene random mutations.

## Introduction

Proteins are made by 20 types of a-amino acids, which have different shapes, dimensions, structures, physicochemical properties [1, 2] and which are observed with different frequencies [3]. Different amino acid properties have been used to predict a variety of protein features, ranging from subcellular location [4] to protein-protein interfaces [5].

Despite its small dimension, this alphabet of 20 characters allowed Nature to create a large numsber of different proteins, amongst the astronomic number of possible sequences that riches the value of 20^N^, where N is the sequence length. Interestingly, protein sequences cannot be back-traced, in the sense that if the sequence ABCDEFG is observed in Nature, the sequence GFEDCBA is not [6]. This asymmetry amongst the possible sequences can be investigated also at the level of short repeats, for example dipeptides.

Nevertheless, here the problem is a bit different, since Nature was able to use all the possible 400 dipeptides that can be written with an alphabet of 20 characters. This means that any of the 400 dipeptides can be frequently found in proteins. In other words, one should not be looking for the existence of the dipepeptide BA, given the existence of the dipeptide AB. As a consequence, the question can be reformulated as follows: is the dipeptide AB equally frequent than the dipeptide BA?

Interestingly, we observed that, in some cases, one of the dipeptides (the AB) is considerably more abundant than its symmetry related antidipeptide BA. The natural abundance of both the amino acids A and B cannot influence the preference of Nature for the dipeptide AB or for the dipeptide BA. We therefore examined a wide series of possible features that might distinguish the dipeptide AB from its counterpart BA. On the one hand, we considered structural features, like secondary structure, accessibility to the solvent or conformational flexibility, and, on the other hand, we examined the possibility that random nucleotide mutations of the genes might cause the prevalence of one of the members of the dipeptide-antidipeptide pair. We did not find any feature that can explain why a certain dipeptide is preferred by Nature over its antidipeptide mirror image. We therefore propose that either this asymmetric frequency is barely casual or that a not yet understood reason determines the occurrence of certain types of dipeptides.

## Methods

### Asymmetric frequency

The comparisons between the 190 pairs of dipeptides AB and BA, where A and B are one of the twenty types of amino acids and A ≠ B, were performed with the quantity *C*_*190*_

$$C_{190}=\frac{|n_{\text{AB}}-n_{\text{BA}}|}{\left(n_{\text{AB}}+n_{\text{BA}}\right)/2}$$

where *n*_*AB*_ and *n*_*BA*_ are the numbers of dipeptides AB and BA. The value of *C*_*190*_ is equal to zero if *n*_*AB*_ = *n*_*BA*_ or, in other words, when the frequency of observation of the dipeptide AB is equal to the frequency of observation of the dipeptide BA. On the contrary, if one of the two dipeptides, for example AB, is observed more frequently than the other (BA), the value of *C*_*190*_ is larger than 0 and it increases if the difference between *n*_*AB*_ and *n*_*BA*_ increases. It is possible to compute 190 values of *C*_*190*_ in a set of protein structures, since both A and B indicate only one type of amino acid and since the dipeptides of identical residues (for example AA, CC, DD etc.) are ignored.

### Propensity

Alternatively, the propensities of a certain type of amino acid to be followed by another type of amino acids were computed. For example, the propensity of alanine to precede glycine is given by

$$P(\text{AG})=\frac{n_{\text{AG}}/n_{\text{XG}}}{n_{\text{AX}}/n_{\text{XX}}}$$

where *n*_*AG*_ is the number of times an alanine precedes a glycine, *n*_*XG*_ is the number of times a residue (of any type) precedes a glycine, *n*_*AX*_ is the number of times an alanine precedes a residue (of any type), and *n*_*XX*_ is the number of times a residue (of any type) precedes a residue (of any type). Note that *n*_*XX*_ is the number of dipeptides observed in the set of protein sequences, *n*_*AX*_ is the number of dipeptides where the first residue is an alanine, *n*_*XG*_ is the number of dipeptides where the second residue is a glycine, and *n*_*AG*_ is the number of alanine-glycine dipeptides. More in general, the propensity of occurrence of a dipeptide BJ is given by

$$P(\text{BJ})=\frac{n_{\text{BJ}}/n_{\text{XJ}}}{n_{\text{BX}}/n_{\text{XX}}}$$

where *n*_*BJ*_ is the number of dipeptides of type BJ, *n*_*XJ*_ is the number of time a residue (of any type) precedes a residue J, *n*_*BX*_ is the number of time a residue B precedes a residue (of any type), and *n*_*XX*_ is the number of times a residue (of any type) precedes a residue (of any type).

### Datasets

Several sets of protein sequences were considered. In all cases, the data were downloaded from the UniProt database and the redundancy was reduced to 40% of sequence identity with the program cd-hit. In each case, only the sequence of entire proteins were taken into account (protein fragments were ignored) and only proteins, the existence of which was proven experimentally, were considered. The datasets are summarized in Table 1.

**Table 1:** List of the ensembles of protein sequences used in the present study.

Dataset	Description	Number of proteins / Number of residues	Notes
Any	Any protein	39,029/19,363,703	
*H.sapiens*	Proteins of *Homo sapiens*	10,290/6,690,249	
*E.coli*	Proteins of *Escherichia coli*	204/63,809	
Mono	Monomeric proteins	1,307/542,334	
Homo	Proteins chains that form homo-oligomeric complexes	3,374/1,455,147	
Hetero	Proteins chains that form hetero-oligomeric complexes	1,490/721,979	
Cyto	Cytoplasmic proteins	9,421/5,622,499	Only proteins the subcellular location of which was proven experimentally
Memb	Membrane proteins	8,757/5,022,480	Only proteins the subcellular location of which was proven experimentally
Extra	Secreted / Extracellular proteins	344/295,553	The subcellular location was proven experimentally

### Techniques

Molecular dynamics were performed in vacuo with the program Dynamic of the Tinker software package (10,000 dynamic steps of 1 femtosecond at 298 Kelvin degrees with the amber99 force field and by recording a model every 0.1 Picoseconds) [7]. Five initial conformations were selected for each dipeptide, the termini of which were not capped, and five simulations were performed for each dipeptides. Results were statistically indistinguishable.

Protein threedimensional structures were extracted from the Protein Data Bank [8, 9] and their redundancy was reduced with PISCES [10]. Secondary structures were assigned with Stride [11] and solvent accessible surface area values were computed with Naccess [12].

## Results and Discussion

### C190 analysis

**Figure 1:**
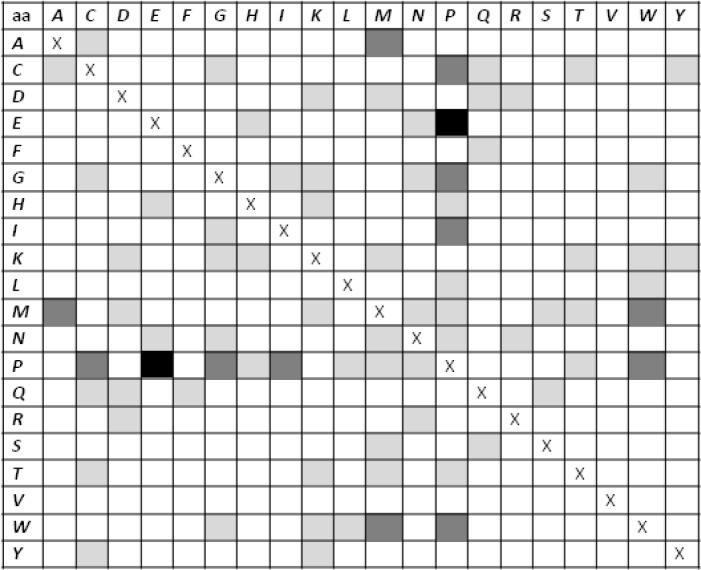
*C*_*190*_ values for the dipeptides AB with (A≠ B). Values are colored according to the following scheme: white if *C*_*190*_ ≤ 10, light gray if 10 < *C*_*190*_ ≤ 20, dark gray if 20 < *C*_*190*_ ≤ 30, and black if *C*_*190*_ > 30.

The *C*_*190*_ values are summarized graphically in Figure 1. Most of them are close to zero, as it must be expected for proteins that contain the same number of dipeptide pairs AB and BA, though some of them are considerably larger than zero. They range from 0.04, for the dipeptides PR/RP, to 33.76, for the dipeptides EP/PE, and their average value is equal to 6.50 (standard error = 0.29).

The twenty average *C*_*190*_ values for the dipeptides that contain one of the twenty types of amino acids are shown in Table 2. It can be seen that if the dipeptides contain proline the *C*_*190*_ values tend to be, on average, higher than the others (average *C*_*190*_ = 11.86). This might be related, to a first approximation, to the conformational rigidity of this particular amino acid, the side chain of which is conjugated on its main chain nitrogen atom. It is possible, in other words, that the rigidity of proline makes it difficult for some residues to precede or to follow it. However, it must be observed that the lowest *C*_*190*_ value is observed for the dipeptides PR/RP, which contain proline and, therefore, any interpretation uniquely based on the fact that proline is conformationally anomalous is likely to be rather naïve. Moreover, in some cases, it is the dipeptide with proline in the first position (PX) that is observed more frequently than the other dipeptide where proline occupies the second position (XP).

**Table 2:** Average *C*_*190*_ values for the didpetides that contain the amino acid X and another one, different from X. Standard errors of the average values are given in parentheses.

X	*C*_*190*_
A	9.03(1.45)
C	9.86(1.87)
D	9.76(1.74)
E	5.21(0.84)
F	4.17(0.73)
G	4.95(1.01)
H	4.77(1.09)
I	5.57(1.14)
K	7.60(1.58)
L	5.65(0.89)
M	10.45(1.53)
N	7.12(1.19)
P	11.86(2.23)
Q	6.02(0.83)
R	4.51(0.80)
S	5.19(0.94)
T	4.89(1.10)
V	3.47(0.72)
W	8.43(1.64)
Y	4.80(0.85)

The second highest average *C*_*190*_ value is associated with the dipeptides that contain methionine. In this case, one must observe that the dipeptides MX are considerably more numerous (789,224) than the antidipeptides XM (717,205) and, as a consequence, the *C*_*190*_ value for the MX/XM pair is much larger the zero (10.45). However, this is certainly due to the highly frequent N-terminal methionines, which are sometime (but not always) retained in the sequences deposited in the databases [13].

High average *C*_*190*_ values are also observed for dipeptide/antidipeptide pairs that contain a particular residue like cysteine (average *C*_*190*_ = 9.86), a residue with an anionic side chain like aspartate (average *C*_*190*_ = 9.76), a small apolar residue like alanaine (average *C*_*190*_ = 9.03), or a large aromatic residue like triptophane (average *C*_*190*_ = 8.43). On the contrary, the smallest average *C*_*190*_ values are observed for the peptide/antidipeptide pairs that contain an apolar amino acid like valine (3.47) of an aromatic residue like phenylalanine (4.17).

Some of the *C*_*190*_ values are certainly large (see Table 3). For example, it is much more common to observe the dipeptide PE (7571 observations) than its antidipeptide counterpart (5384 observations) and the *C*_*190*_ of the PE/EP pair is equal to 33.76. Seven pairs of dipeptide/antidipeptide have a *C*_*190*_ larger than 20 (see Table 3). Five of them involve proline and the other two methionine. The other residues can be large (triptophane) or small (glycine and alanine). In only one pair of dipeptide/antidipeptide there is a polar amino acid (glutamic acid). Interestingly, also the pair GP/PG, which contains the two residues (proline and glycine) that are conformationally most different from all the other 18 amino acids, has one of the highest *C*_*190*_ values.

**Table 3:** The seven pairs of dipeptide/antidipeptide with the highest *C*_*190*_ values.

dipeptide	n. observations	antidipeptide	n. observations	*C*_*190*_
EP	5384	PE	7571	33.76
PW	1113	WP	873	24.14
MW	403	WM	513	24.12
GP	5965	PG	7486	22.61
AM	2933	MA	3652	21.85
IP	5417	PI	4384	21.07
CP	1840	PC	1490	20.97

### Sampling tests

In order to verify if these trends are genuine or are a simple consequence of the insufficient sampling of the protein sequences, I adopted two strategies.

On the one hand, the *C*_*190*_ values were computed on different sets of proteins (see Table 1). I considered proteins expressed in a single organism (*Homo sapiens* and *Escherichia coli*), localized in a single sub-cellular location (cytoplasm, membrane, extracellular space), or adopting different types of quaternary structure (monomeric, homooligomeric, and heterooligomeric proteins). The *C*_*190*_ values computed with all these different datasets are shown in Table 4. Several oscillations are observed amongst the different sets. For example, the *C*_*190*_ value for the dipeptides/antidipeptide pair CP/PC ranges from 12 (in the set of membrane proteins) to 30 (in the set of *E. coli* proteins). However, the *C*_*190*_ values of the dipeptides shown also in Table 3 are always much larger than zero. This supports the hypothesis that the trends previously described are genuine and do not depend on the fact that the amount of information is insufficient. In other words, it is possible to be quite confident that the number of protein sequences used to compute the *C*_*190*_ values is sufficient to delineate a statistically significant tendency.

**Table 4:** *C*_*190*_ values computed with the sequence sets summarized in Table 1. Only the *C*_*190*_ values for the seven pairs of dipeptide/antidipeptide of Table 3 are reported.

	*H.sapiens*	*E.coli*	Cyto	Memb	Extra	Mono	Homo	Hetero
*C*_*190*_(EP/PE)	30.31	59.39	34.76	31.27	15.11	45.96	37.5	34.92
*C*_*190*_(PW/WP)	27.51	31.25	26.54	26.27	31.06	8.79	29.19	41.51
*C*_*190*_(MW/WM)	19.13	20.5	21.47	20.02	23.73	50.6	30.46	42.49
*C*_*190*_(GP/PG)	20.46	50	18.33	19.76	31.01	29.18	27.08	23.31
*C*_*190*_(AM/MA)	32.82	31.58	18.46	19.27	48.23	27.86	22.5	31.53
*C*_*190*_(IP/PI)	29.93	52.48	23.42	23.22	15.21	29.24	20.12	25.37
*C*_*190*_(CP/PC)	19.69	30.3	15.72	12.18	17.11	17.77	23.92	27.23

On the other hand, I used an approach named the Fragmented Prediction Performance Plot [14]. The *C*_*190*_ values were computed by using smaller datasets of increasing size. First, I used 39 non-overlapping subsets, taken from the Any dataset of Table 1 and each containing 1,000 proteins, and the averages of the *C*_*190*_ values were computed, together with their standard deviations. Then the same procedure was applied a second time to 13 non-overlapping subsets of 3,000 proteins. And then, a third time, with six subsets of 6,000 proteins. And eventually, a fourth time, by using two non-overlapping subsets of 12,000 proteins. Some relevant results are summarized in Table 5. If there were large oscillations amongst the *C*_*190*_ values computed with sets of different sizes, one would conclude that the amount of information is insufficient and that no reliable and significant values of *C*_*190*_ can be computed. On the contrary, if the *C*_*190*_ values were constant and independent of the dimension of the subsets, one would conclude that the number of proteins is sufficient to make reasonable esstimations of the *C*_*190*_ values. In Table 5 it is possible to see that the values of *C*_*190*_ are rater independent on the number of protein sequences used to compute them. The same is true also for the other *C*_*190*_ values that are not shown in Table 5. As a consequence, it is possible to be quite confident that the number of protein sequences used to compute the *C*_*190*_ values is sufficient to delineate a statistically significant tendency.

**Table 5:** *C*_*190*_ values computed with protein subsets of increasing size from 1000, to 3000, to 6000, to 12000 proteins until all the sequences are included into the analysis. Standard errors are given in parentheses.

	1,000	3,000	6,000	12,000	all
*C*_*190*_(PE/EP)	34.29(0.90)	33.99(1.22)	33.89(1.28)	33.65(0.62)	33.76
*C*_*190*_(PW/WP)	23.81(2.04)	23.57(2.65)	23.59(1.39)	23.71(0.25)	24.14
*C*_*190*_(MW/WM)	25.11(2.53)	23.52(3.28)	23.78(3.21)	23.78(0.57)	24.12
*C*_*190*_(GP/PG)	23.03(1.21)	22.28(0.86)	22.17(0.69)	22.13(0.58)	22.61
*C*_*190*_(AM/MA)	21.82(1.16)	22.11(1.04)	21.98(1.14)	22.06(0.91)	21.85
*C*_*190*_(IP/PI)	21.11(0.90)	21.16(0.90)	21.05(0.78)	21.13(0.68)	21.07
*C*_*190*_(CP/PC)	22.69(1.46)	21.65(1.54)	20.82(1.24)	20.83(0.96)	20.97

### Propensities

Table 6 shows the seven pairs of peptide/antipeptide that have the largest difference in propensity. It can be immediately seen that these seven pairs are the same of the seven pairs of Table 3, with the exception of the pair EN/NE which is replaced in Table 3 by the pair AM/MA. The propensity values agree therefore with the *C*_*190*_ values and it can be concluded that (i) there are some dipeptides that are observed much more (or less) frequently than their corresponding antidipeptides; (ii) often proline is part of these dipeptides/antidipeptides; (iii) the GP/PG pair, that contains both the residue with anomalous Ramachandran plots, is amongst the dipeptides that behave differently from their antidipeptide counterparts.

**Table 6:** The seven pairs of dipeptide/antidipeptide with the largest difference in propensity of a residue to be followed by another residue. In the first line, for example, it can be read that the propensity of a methionine to be followed by a triptophane in equal to 0.83 while the propensity of a triptophane to be followed by a methionine is 1.17.

Dipeptide	Propensity	Antidipeptide	Propensity	Difference
MW	0.83	WM	1.17	0.34
PE	1.05	EP	0.75	0.3
GP	0.89	PG	1.12	0.23
CP	1.08	PC	0.87	0.21
PW	0.93	WP	0.73	0.2
EN	1.1	NE	0.9	0.2
IP	1	PI	0.81	0.19

### Neighbor effects?

The fact that a dipeptide is more (or less) frequent that its antidipeptide counterpart can depend on numerous factors. The most obvious is that the non-bonding interactions between residue A and residue B in the dipeptide AB are different from those in the dipeptide BA. It is possible that the conformational space accessible to AB is different from that accessible to BA. In other words, two dipeptides of opposite sequence might have an anisotropic conformational energy.

A first way to test this hypothesis is to compute *C*_*190*_ and propensity values for the dipeptides A(X)_n_B and B(X)_n_A. In these dipeptides, the residues A and B are separated by *n* other residues (of any type). To a first approximation, if *n* is sufficiently large, the residues A and B cannot interact with each other in these dipeptides. However, it is advisable to avoid large values of *n*, which would reduce the number of dipeptides that can be analyzed (for example, in a protein containing *n*+2 amino acids, there is only one A(X)_n_B dipeptide). For these reasons, the value of *n* was fixed at 5. This value is sufficiently large to avoid inter-residue contacts (and interactions), even in alpha-helical segments, and small enough to allow the formation of large sets of data.

*C*_*190*_ and propensity values for these B(X)_n_A/A(X)_n_B pairs are shown in Table 7. It is apparent that the *C*_*190*_ values, even if yet quite different from 0, are much smaller than the values of Table 3. Moreover, the propensity values tend to converge, in the sense that they are nearly identical for the B(X)_n_A and A(X)_n_B dipeptides. It can therefore be concluded that if there are five residues between the two amino acids A and B, the reciprocal influence between residue A and residue B is extremely much smaller. The anisotropic frequency of the AB and BA dipeptides seems therefore strictly related to short range and geometrically local inter-residue interactions.

**Table 7:** Analysis of the pairs of dipeptide/antidipeptide (AB/BA) reported in Tables 3 and 6 in the form of A(X)_5_B, when five amino acids (of any type) are intercalated between A and B.

Dipeptide / antidipeptide	*C*_*190*_	Prop. Dipept.	Prop. Antidip.	Difference
EP/PE	2.74	0.9	0.87	0.03
PW/WP	2.65	1.04	1.02	0.02
MW/WM	3.71	0.96	1.02	0.06
GP/PG	3.85	1.02	0.98	0.04
AM/MA	10.13	1	1	0
IP/PI	3.94	0.89	0.92	0.03
CP/PC	0.21	1.03	1.02	0.01
EN/NE	2.81	0.92	0.95	0.03

### Reification attempts

The different occurrence of dipeptides and antidipeptides may result from physicochemical reasons or from genetic evolution.

In the first case, one might verify if the physico-chemical properties of the dipeptide AB are different from those of the dipeptide BA. Moreover, this must be done not only for the pairs AB/BA that show a relevant asymmetry of occurrence but also on the pairs XY/YX that show the same frequency of occurrence. In fact, in this way, it is possible to try to discover if the different occurrence of a dipeptide/antidipeptide pair is due to physico-chemical causes.

For this reason, a series of comparison were made between the dipeptides shown in Tables 3 and 6 (EP/PE, PW/WP, MW/WM, GP/PG, AM/MA, IP/PI, CP/PC, EN/NE), which have a different frequency of occurrence, and the results were compared to the molecular dynamics simulations of another set of pairs of dipeptides (AR/RA, CD/DC, DV/VD, EY/YE, FT/TF, HT/TH, PR/RP, ST/TS) that did not show any difference in their *C*_*190*_ values or in their propensity values. These comparison were performed on a non redundant set of 1758 protein crystal structures (maximal pairwise sequence identity = 20%, crystallographic resolution not worse than 1.6 Å and R factor not worse than 0.25) created with the PISCES web server [10] and where structures with missing atoms or residues (a phenomenon much more common that usually thought [15]) were disregarded.

The secondary structures, assigned with the Stride computer program [11], the atomic displacement parameters, normalized to zero mean and unit variance in order to allow one to compare different crystal structures [16, 17], and the solvent accessibilities, monitored with the Naccess software [12], were unable to distinguish the two types of peptide/antidipeptide pairs. Similarly, a serried of molecular dynamics simulations did not show a different behavious amongst the two types of dipeptide/antidipeptide pairs. Similarly, it was observed that none of the dipeptide/antidipeptide pairs examined here have a systematic tendency to be located at the borders of any type of secondary structural element.

Another possible reification of the asymmetric frequency of certain peptide/antidipeptide pairs relies on gene sequences. It is possible that certain dipeptides are more frequent than others because of the different probability of their emergence as a consequence of nucleotide deletions/mutations. To test this hypothesis, the sequences of the human genes available at the RefSeq database were considered (ftp://ftp.ncbi.nih.gov/refseq/). For each of them, one hundred mutants were created by randomly deleting one of the bases, one hundred mutants were built by deleting randomly five bases, one hundred mutants were generated by randomly mutating ten bases, and one hundred mutants were made by changing randomly fifty bases.

After translation of the sequences, performed with the program Transeq of the EMBOSS software suite [18], the *C*_*190*_ values were computed together with the propensities. These were identical in the wild type sequences and in all the four types of mutants. It seems therefore reasonable to suppose that random modifications at the genic level are not responsible for the fact that some dipeptides are more frequent in proteins than others.

## Conclusions

Some dipeptides are considerably more frequent than others in proteins. This was quantified by means of two figures of merit, the *C*_*190*_ and the propensity, which monitors different features. The first (*C*_*190*_) monitors to which extent a dipeptide AB is more common than its antidipeptide counterpart BA. The second (propensity) is on the contrary a measure of probability and it indicates the tendency of B to follow A in the dipeptide AB (or the tendency of A to follow B in the antidipeptide BA). Although they are based on different models, both the values of *C*_*190*_ and of propensity indicate that some dipeptides are much more common than their antidipeptides (see Tables 3 and 6).

This does not seem to be caused by insufficient sampling. An FPPP analysis [14] shows that the amount of data is sufficient to delineate reliable trends. Moreover, similar tendencies were observed on smaller and more homogeneous sets of protein sequences (monomeric, homooligomeric, heterooligomeric, human, bacterial, nuclear, cytoplasmic or extracellular).

Despite numerous attempts, it has been impossible to identify the reasons that make some dipeptides much more common than their mirror images. Local conformational flexibility and local structures were found to be unrelated to the dipeptide frequency as well as the degree of solvent exposure. Also genic mutations were found to be independent of the dipeptide rate of occurrence.

Although it is reasonable to suppose that the intrinsic structural and molecular properties of dipeptides are determined by both their intermolecular connectivity and their interactions with the surrounding environment (see for example the thorough studies on the structures of several dipeptides and on the influence of the solvatation [19, 20]), the reasons why some dipeptides are considerably more frequent than their antidipeptide counterparts remains, for the moment, elusive and obscure. This phenomenon is however very surprising and would deserve further analyses in the future.

In particular, one can anticipate that analyses on longer protein segments (like for example tripeptides, tetrapeptides or longer peptides) might provide additional and interesting information. Unfortunately, the information presently available in the databases, especially about protein structures, is insufficient to perform reliable statistical surveys of these longer polypeptide fragments. It is also possible that additional and interesting information might be provided by more extensive molecular dynamics simulations of the dipeptide/antidipeptide pairs, both isolated and in the context of protein structures. Eventually, a further open question is the understanding of why some residues prefer to precede of follow other residues, something that can be examined by considering the sign, positive or negative, of the *C*_*190*_ values, in analogy with what is done with the propensity values.

## References

[1] Gromiha MM (2010) Protein bioinformatics: From sequence to structure Amsterdam: Academic Press

[2] Gasteiger E, Hoogland C, Gattiker A, Duvaud S, Wilkins MR, et al. (2005) Protein Identification and Analysis Tools on the ExPASy Server In: Walker JM, editor The Proteomics Protocols Handbook: Humana Press pp. 571–607

[3] Carugo O (2008) Amino acid composition and protein dimension. Protein Sci 17: 2187–2191 18780815 10.1110/ps.037762.108 PMC2590925

[4] Mei S, Fei W (2010) Amino acid classification based spectrum kernel fusion for protein subnuclear localization. BMC Bioinformatics 11: S17 20122188 10.1186/1471-2105-11-S1-S17 PMC3009488

[5] Zellner H, Staudigel M, Trenner T, Bittkowski M, Wolowski V, et al. (2012) PresCont: predicting protein-protein interfaces utilizing four residue properties. Proteins 80: 154–168 22038731 10.1002/prot.23172

[6] Carugo O (2010) Structural similarity between native proteins and chimera constructs obtained by invertinf the amino acid sequence. Acta Chim Slov 57: 936–940 24061900

[7] Ren P, Wu C, Ponder JW (2011) Polarizable Atomic Multipole-Based Molecular Mechanics for Organic Molecules. J Chem Theory Comput 7: 3143–3161 22022236 10.1021/ct200304d PMC3196664

[8] Bernstein FC, Koetzle TF, Williams GJ, Meyer EF, Jr., Brice MD, et al. (1977) The Protein Data Bank: a computer-based archival file for macromolecular structures, J Mol Biol 112: 535–542 875032 10.1016/s0022-2836(77)80200-3

[9] Berman HM, Westbrook J, Feng Z, Gilliland G, Bhat TN, et al. (2000) The Protein Data Bank. Nucleic Acids Res 28: 235–242 10592235 10.1093/nar/28.1.235 PMC102472

[10] Wang G, Dunbrack RLJ (2005) PISCES: recent improvements to a PDB sequence culling server. Nucleic Acids Res 33: W94–W98 15980589 10.1093/nar/gki402 PMC1160163

[11] Frishman D, Argos P (1995) Knowledge-Based Protein Secondary Structure Assignment Proteins: Structure, Function, and Genetics. Proteins 23: 566–579 8749853 10.1002/prot.340230412

[12] Hubbard SJ, Campbell SF, Thornton JM (1991) Molecular recognition. Conformational analysis of limited proteolytic sites and serine proteinase protein inhibitors. J Mol Biol 220: 507–530 1856871 10.1016/0022-2836(91)90027-4

[13] Giglione C, Boularot A, Meinnel T (2004) Protein N-terminal methionine excision. Cell Mol Life Sci 61: 1455–1474 15197470 10.1007/s00018-004-3466-8 PMC11138929

[14] Carugo O (2007) Detailed estimation of bioinformatics prediction reliability through the Fragmented Prediction Performance Plots. BMC Bioinformatics 8: 380 17931407 10.1186/1471-2105-8-380 PMC2148069

[15] Carugo O (2011) Participation of protein sequence termini in crystal contacts, Protein Sci 20 10.1002/pro.690 PMC3302655 21739502

[16] Carugo O, Argos P (1999) Reliability of atomic displacement parameters in protein crystal structures. Acta Crystallogr D55: 473–478 10.1107/s0907444998011688 10089358

[17] Carugo O, Argos P (1997) Correlation between side chain mobility and conformation in protein structures. Protein Eng 10: 777–787 9342144 10.1093/protein/10.7.777

[18] Rice P, Longden I, A. B EMBOSS: (2000) The European Molecular Biology Open Software Suite, Trends Genet 16 10827456 10.1016/s0168-9525(00)02024-2

[19] Das G (2013) Investigations of dipeptide structures containing pyrrolysine as N-terminal residues: a DFT study in gas and aqueous phase. J Mol Model 19: 1901–1911 23334349 10.1007/s00894-013-1754-7

[20] Mandal S, Das G (2013) Structure of dipeptides having N-terminal selenocysteine residues: a DFT study in gas and aqueous phase, J Mol Model in the press. 10.1007/s00894-013-1808-x 23494524

